# Remote Stimulation of Sciatic Nerve Using Cuff Electrodes and Implanted Diodes

**DOI:** 10.3390/mi9110595

**Published:** 2018-11-14

**Authors:** Arati Sridharan, Sanchit Chirania, Bruce C. Towe, Jit Muthuswamy

**Affiliations:** School of Biological & Health Systems Engineering, Ira A. Fulton School of Engineering, Arizona State University, Tempe, AZ 85287, USA; asridhar@asu.edu (A.S.); Sanchit.Chirania@asu.edu (S.C.); bruce.towe@asu.edu (B.C.T.)

**Keywords:** wireless, implantable, microstimulators, neuromodulation, peripheral nerve stimulation, neural prostheses, microelectrode, neural interfaces

## Abstract

We demonstrate a method of neurostimulation using implanted, free-floating, inter-neural diodes. They are activated by volume-conducted, high frequency, alternating current (AC) fields and address the issue of instability caused by interconnect wires in chronic nerve stimulation. The aim of this study is to optimize the set of AC electrical parameters and the diode features to achieve wireless neurostimulation. Three different packaged Schottky diodes (1.5 mm, 500 µm and 220 µm feature sizes) were tested in vivo (*n* = 17 rats). A careful assessment of sciatic nerve activation as a function of diode–dipole lengths and relative position of the diode was conducted. Subsequently, free-floating Schottky microdiodes were implanted in the nerve (*n* = 3 rats) and stimulated wirelessly. Thresholds for muscle twitch responses increased non-linearly with frequency. Currents through implanted diodes within the nerve suffer large attenuations (~100 fold) requiring 1–2 mA drive currents for thresholds at 17 µA. The muscle recruitment response using electromyograms (EMGs) is intrinsically steep for subepineurial implants and becomes steeper as diode is implanted at increasing depths away from external AC stimulating electrodes. The study demonstrates the feasibility of activating remote, untethered, implanted microscale diodes using external AC fields and achieving neurostimulation.

## 1. Introduction

Neuromodulation for peripheral nerve stimulation (PNS) applications is increasingly being used to treat and manage chronic diseases (i.e., epilepsy, micturition, pain, etc. [1,2,3,4]). A major problem with chronic neurostimulation of peripheral nerve for purposes of neural interfacing is that of lead wires tugging on microelectrodes penetrating into the body of a nerve. This becomes a more severe problem when large numbers of wires are used for advanced multichannel neural interface systems needed for both sensory and motor control of prosthetics [5]. Such systems require more channels than can be provided by most nerve cuff systems and need to contact or stimulate nerves lying deeper at the fascicular and subfasicular level. Penetrating needle electrode arrays such as the Utah array (USEA), flat interface nerve electrodes (FINE), transverse intrafascicular multi-channel electrodes (TIME), and longitudinal intrafascicular electrodes (LIFE) [6,7], are increasingly being used to make this connection but lead wire ribbon cables create differential inertia during sudden movement and the potential for damaging nerves during normal nerve movement with the limb. Wireless systems using RF, optical, heat, magnetic and ultrasound energy are increasingly being considered for neuromodulation [8,9,10,11,12,13,14,15]. The present work suggests the potential use of free-floating, stimulating, diode-electrode systems that are wholly implanted within the nerve and the use of strong electric field gradients produced by extraneural electrodes to achieve channel selection.

Excitable tissues of the body are not generally stimulated by short pulses of zero-mean, high frequency (>100 kHz) electric alternating currents (AC) at typically used amplitudes (10 µA–10’s of mA). In fact, classic strength-duration curves reflect that nerve excitation at lower durations (corresponding to high frequency) of stimulus require exponentially increasing monophasic current amplitudes for stimulation. Recent nerve stimulation studies using transdermal amplitude modulated signals (TAMS) using computational models and in vivo experiments indicate that sinusoidal carrier waves of frequencies >20 kHz (variable amplitudes) do not significantly enhance the activation of neurons [16].

However, it is known that high-frequency, pulsed, monophasic (half wave-rectified) or partially rectified currents can stimulate a nerve and do so in ways that depend more on the envelope of the pulse rather than its carrier frequency [17]. Such stimulation currents can be achieved by diodes that rectify high frequency AC currents driven in tissues by remote electrodes that behave according to induced field distributions of volume conductors. Diodes placed in tissue rectify the fraction of high-frequency currents that pass through them relative to that passing through the tissue and can cause local neural activation, as we demonstrate in this study. Different diode placements on the nerve elicited selective electromyogram (EMG) responses in different muscle groups. The differential motor responses suggest the potential for the employment of many very small diodes dispersed around and within nerve to achieve a multichannel configuration driven by combinations of remote electrodes.

This approach to using volume-conducted currents to power implanted diodes and other devices in tissue was first explored by Palti [17] and others more recently [18,19,20,21] for direct stimulation of muscles [18] and nerves [19,20,21]. But a careful assessment of nerve activation using smaller microscale diodes as a function of the AC stimulation parameters such as frequency, peak-to-peak voltage amplitudes and diode parameters such as diode–dipole lengths, feature size and relative position of the diodes with respect to the stimulation electrodes has not yet been done. We find that non-stimulatory AC currents can be remotely driven in the conductivity of the nerve and a small diode with attached microelectrodes will allow intra-neural placement. In this situation, there is the potential for highly localized neurostimulation because of the short dipole spacing of the electrodes on the diode. Therefore, the key aspects of this design are the electric field gradient in tissue and the geometric factors of the diode and its electrodes.

The effect of diodes in a volume-conducted AC field has been modeled for various dipolar configurations in prior modeling studies [22,23]. This study models specific geometric relationships between the diode’s electrodes, the remote activation electrodes, their proximity, and orientation. There is particularly a strong dependence on the anode–cathode length of the diode and the distance of remote activation electrodes. In general, volume conduction of significant amounts of current is limited by the roughly cubic expanding region of reduced current density around the stimulation electrodes as a function of separation distance. Even so, relatively high amplitude pulsed AC currents at high frequencies are well tolerated by tissues and so offer a way to help overcome path losses. We investigate the potential for energy transfer within constraints acceptable for local power transfer from outside a nerve epineurium to inside the nerve. The focus of the study here is to partly understand the limitations on the scheme of placing very short, but untethered diode–dipoles within a nerve cuff and then using differences in volume conduction and electrode current path lengths to define diode activation. This is proposed in order to reduce the need for penetrating electrodes and their potential for damage by lead wires. It is this key aspect of ‘wirelessness’ at this point in the chain of electrical pulse generators and lead wires that offers an advantage to the development of advanced nerve interfaces. The concept we are testing is the potential to achieve multichannel stimulation of a compound nerve by inserting or placing multiple small diodes within or on the nerve such that each responds to specific combinations of remote electrodes providing different configurations of electrical fields. Such diodes will not be connected by wires. This concept is enabled for experimentation by the availability of commercially available Schottky diodes in formats such as the Skyworks CDC-7630 having a 1.5 mm length and unpackaged silicon diode die of 220 μm square, but we note that diodes are easily made by modern photolithography at much smaller sizes.

## 2. Materials and Methods

### 2.1. In Vivo Rodent Sciatic Nerve Model

All animal procedures were done with the approval of the Institute of Animal Care and Use Committee (IACUC) of Arizona State University and in accordance with the National Institute of Health (NIH) guidelines. All efforts were made to minimize animal suffering and to use only the number of animals necessary to produce reliable scientific data. In all, 17 rats were used in total for all experiments.

Briefly, 300–600 g male Sprague–Dawley (*Rattus norvegicus*) rats (*n* = 17 rats total) were anesthetized (induction) using 50 mg/mL ketamine, 5 mg/mL xylazine, and 1 mg/mL acepromazine administered via intraperitoneal injection and maintained with 0.5–1% isoflurane. The left hind legs were shaved and residual hair was removed using hair removal cream. The animal was mounted on a stereotaxic frame and heart rate (~280–350 beats/min) and breathing (~60 breaths/min) were monitored using SurgiVet™ (Smith Medical Systems, Dublin, OH, USA). Aseptic techniques to disinfect the skin (i.e., application of isopropyl alcohol or betadiene) were used to ensure sterility. After skin incision and dissection of the muscle planes, the sciatic nerve was identified and isolated. Connective tissue surrounding the nerve was gently removed using iris microscissors at least 1 cm distal from the trifurcation point. The nerve cuff described previously was placed approximately 1 cm distal from the trifurcation point where the sural, peroneal and tibial bundles split. The cuff was placed such that the insulating silicone bottom under the rings as the only contact point with the rat body to ensure no contact with surrounding muscle groups to prevent potential off-target stimulation effects. A total of 10 (out of 17) animals were used for characterizing the performance of remote diodes with a stimulation threshold as a function of AC stimulation parameters (i.e., frequency, AC burst duration, measurements of diode current amplitudes based on relative position of remote diodes from stimulating electrodes, diode–dipole length and implantation depth).

To demonstrate that modified, implanted mini-, and micro-diodes can stimulate the nerve, needle-based electromyography (EMG) was used. Disposable monopolar needle electrodes (Rhythmlink™, Columbia, SC, USA) were placed in digit 5 of the rat hind leg paw (either left or right) for nerve cuff based experiments. The animal was grounded with a needle electrode in the opposite hind leg. EMGs were recorded using Intan™ recording system (Intan, Los Angeles, CA, USA) and analyzed in MATLAB offline. The recordings were digitally filtered on the Intan™ system using a bandpass filter from 100–3000 Hz to remove motion artifacts. EMG recordings were analyzed for 10 repetition trials of each stimulation condition.

Large SC-79 package diodes were also used to test selectivity. Multiple EMG electrodes at different sites (ankle/plantar, biceps femoris, and tibalis anterior) were placed in 3 (out of the total of 17) additional animals to test for muscle selectivity using AC excitation at 300 kHz or 1 MHz. Muscle response was recorded using needle-based EMGs.

Mean ± standard deviation of the EMG amplitude was calculated and muscle recruitment curves were plotted for nerve stimulation using diodes placed subepineurally and diodes implanted in the sciatic nerve. A total of 4 additional animals (out of the total 17) were used for the in vivo validation (2 for subepineurial and 2 for deep nerve implants). For stimulus threshold voltage measurements, mean ± standard error (SE) was plotted and statistical analysis was performed using one-way analysis of variance (ANOVA) and if found significant, the maximum and minimum values were evaluated for significance (α = 0.01) using the Student’s *t*-test.

### 2.2. Diode Packaging and Modification

**Figure 1:**
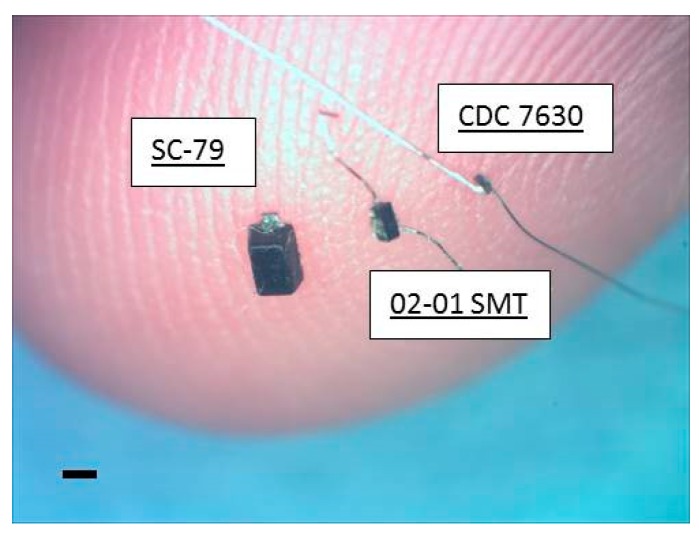
Pictures of the modified Skyworks 7630 diodes placed on an adult (digit 5) finger that were used for wireless neuromodulation. Images of the mini- and micro-diodes are prior to lead trimming and subsequent implantation into the peripheral nerve. The mini-diode is capable of placement under the epineurium, while the micro-diode is capable of both subepineurial and deep nerve tissue placement. Scale bar is 0.5 mm.

Ultra-small, commercially-available Schottky diodes (Skyworks 7630, Woburn, MA, USA) were purchased in three different packages (SC-79, 0201 SMT, and bare die CDC7630)). The diode lengths were 1.5 mm, 0.5 mm and 0.22 mm respectively, with the first diode capable of stimulation on or outside the nerve, while the second and third diode packages offered intraneural, implantable sizes. The implantable 0201 SMT and bare die (hereafter referred to as mini- and micro-diodes respectively) were connected with 50 µm diameter platinum leads for nerve tissue contact that could be trimmed to desired diode–dipole lengths. Mini- and micro-diodes were dipped in a fluorosilane-based coating (3M-Novec EGC-1720) for 2 min and dried at room temperature for insulating electrically sensitive portions of the device. An additional, ethyl-cyanoacrylate based layer (Gorilla™ impact tough super glue) was added to strengthen the bond between the platinum leads and the diode bond pads to withstand mechanical stresses from the animal for durable implantation. The three packaged diodes are shown against the tip of smallest finger of a human hand in Figure 1.

**Figure 2:**
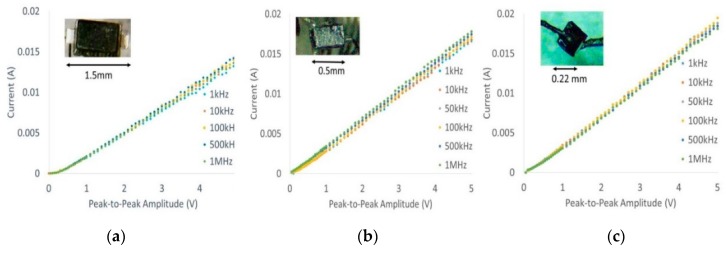
Current–voltage (I-V) characterization of modified, mini- and micro-diode packages—(**a**) SC-79, (**b**) 02-01SMT, (**c**) CDC 7630--for 10 kHz–1 MHz expectedly showed no significant electrical deviations due to frequency. Peak-to-peak, rectified current was measured across a load of 1.1 kΩ resistor.

Current–voltage characteristic (I-V) curves of all three packages shown in Figure 2 were generated (10 kHz–1 MHz) using a Siglent™ function generator and oscilloscope to ensure that post-modifications did not affect diode characteristics such as threshold voltage as a function of frequency. Typical thresholds ranged from 150–180 mV at different frequencies, which is comparable to manufacturer datasheets.

### 2.3. Nerve Cuff Testing Platform

**Figure 3:**
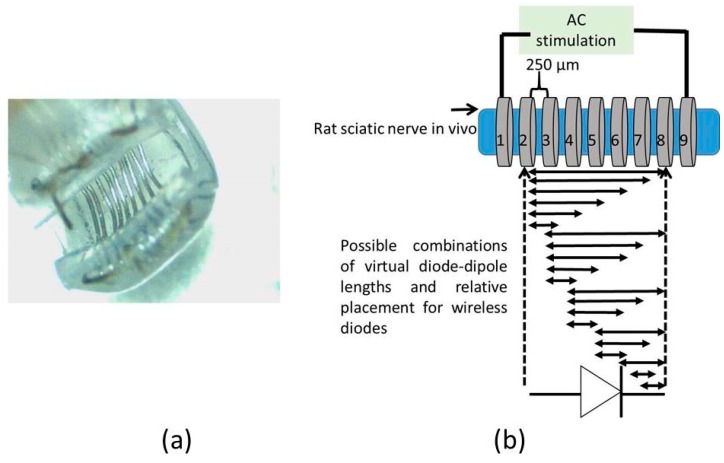
Nerve cuff-based platform was used to test currents through a diode placed superficially on a nerve for different diode–dipole lengths and placement of diodes relative to the external alternating current (AC) excitation voltages. (**a**) Image of a 9-ring nerve cuff with 100 µm diameter platinum leads spaced 250 µm apart. (**b**) Rings ‘1’ and ‘9’ were used to supply AC stimulus drive voltage (10 kHz–1 MHz) on the nerve. Rings ‘2’ through ‘8’ were used to test different combinations of diode–dipole lengths and the position relative to the AC drive voltages.

A cuff-electrode was used as a platform to generate AC fields in the sciatic nerve. A nerve cuff with 100 µm diameter platinum electrodes with 9 rings spaced 250 µm apart as shown in Figure 3a was custom fabricated by Microprobes (Gaithersburg, MD, USA). The total distance between the inner edge of electrode rings ‘1’ and ‘9’ was 2.7 mm. Each ring on the cuff had an impedance of ~2 kΩ at 1 kHz. This set-up was used to measure currents through a diode that is superficially placed on the sciatic nerve between two electrodes with AC excitation.

**Figure 4:**
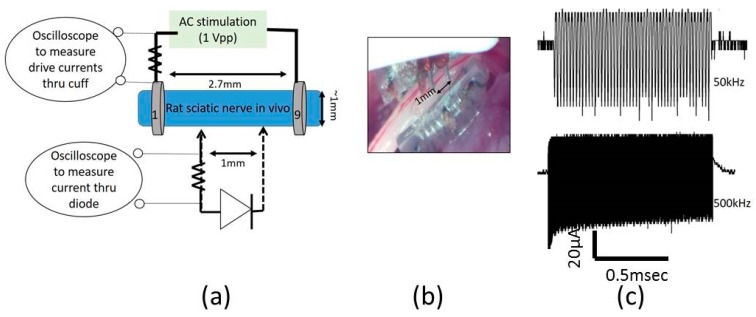
Experimental setup to measure current through remote mini-diodes whose cathode and anode are implanted at different depths (0, ~500 μm, ~1 mm) inside the sciatic nerve. (**a**) An AC stimulus (1 Vpp, peak-to-peak amplitude) is placed on rings ‘1’ and ‘9’ spaced 2.7 mm apart. (**b**) Insulated, platinum microwires (~110 μm diameter) spaced 1 mm apart and externally connected to a mini-diode are placed on the ‘edge’ (~250 μm) away from a stimulus ring electrode or near the ‘center’, which is ~1 mm away from the stimulus electrode. In this image, the probe is placed at the edge. The output is recorded via an oscilloscope and the current is calculated from voltage measurements across a 510 Ω resistor. (**c**) Examples of the partially rectified diode output for 50 kHz and 500 kHz waveforms with 1 msec burst.

**Figure 5:**
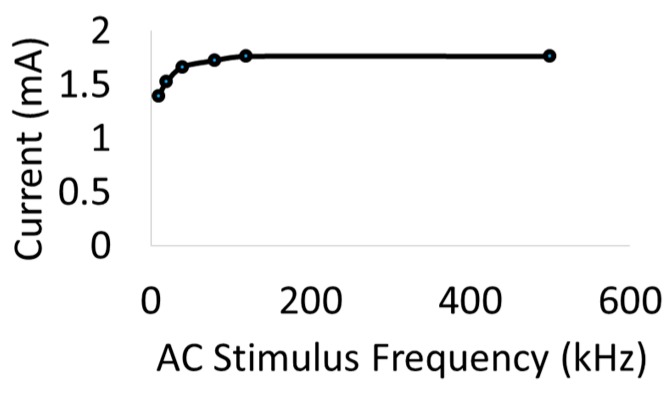
Representative currents measured through a sciatic nerve with a cuff in vivo. Variability in current amplitudes across animals was within ~15%. Currents were measured through a 510 Ω resistor for a 1 Vpeak-to-peak (1 Vpp) input voltage.

To study the impact of diode–dipole length and placement of the diodes relative to the excitation electrodes on nerve excitation, the outer rings ‘1’ and ‘9’ were used to deliver AC stimulation (peak-to-peak voltage of 0–20 V sine waveform) to the sciatic nerve in vivo. The mini- or micro-diode was wired to any 2 of the remaining rings (rings 2 through 8). Different combinations of the inner rings were used to test different diode–dipole lengths with the externally attached, mini- or micro-diode as illustrated in Figure 3b. A 510 Ω resistor was placed in series with the diode for current measurements through the diode and in series with the function generator for current measurements through rings ‘1’ and ‘9’ in the cuff for comparison (Figure 4a). The AC stimulation leads were electrically isolated using a custom-built transformer with a broad frequency range (10 kHz–2 MHz) to prevent ground loops. At 10 kHz, the output was slightly attenuated by 25% and was adjusted in current calculations. Figure 4a shows the setup for drive current measurements with a 510 Ω resistor placed in series with AC input. As seen in Figure 5, the current through the nerve was between 1–2 mA and fairly stable across frequencies. At lower frequencies (10–20 kHz), a marginal dependence on frequency was observed.

To measure typical currents through a microdiode that is implanted in a nerve, the anode and the cathode of the diodes were connected to Teflon-insulated platinum wires (~110 µm diameter, A–M Systems) spaced 1 mm apart as shown in Figure 4a,b. The diode was then mounted on a micromanipulator and the ends of the platinum wires were then used as probes to measure current through different depths in the nerve, namely (a) on the surface of the epineurium, (b) subepineurial placement (c) ~500 µm deep in the sciatic nerve, and (d) ~1 mm deep inside the nerve. Two different diode positions—‘edge’ which is ~250 μm away from the stimulating electrode, and ‘center’ which is 1 mm away from the stimulating electrode)—were assessed for current flow. Examples of partially rectified output for a 1 Vpp (peak-to-peak amplitude) input AC burst at 50 kHz and 500 kHz are shown in Figure 4c.

## 3. Results

### 3.1. Stimulus Voltage Threshold for the Muscle Increases Non-Linearly with Frequency of Alternating Current (AC) Stimulation but the Presence of the Diode Microstimulator on the Nerve Lowers the Stimulus Voltage Threshold

**Figure 6:**
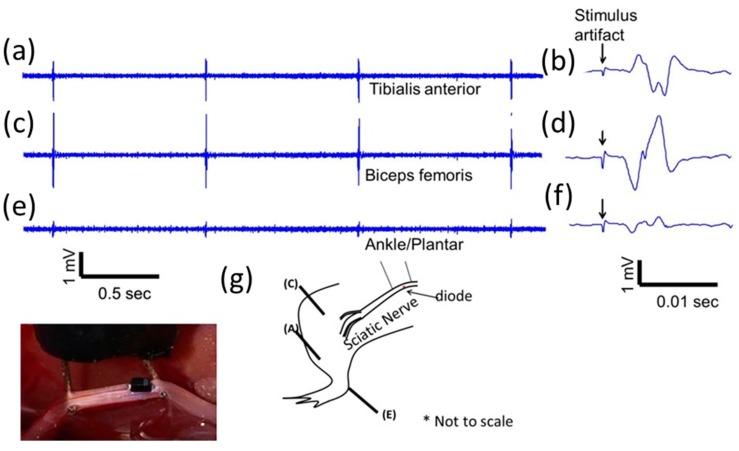
Representative electromyographic (EMG) responses from different muscle groups due to a single, remote, SC-79 packaged Skyworks diode stimulated with a high frequency (1 MHz) AC field by platinum hook electrodes. (**a**,**c**,**e**) are EMG responses at 1 Hz repetition rate. (**b**,**d**,**f**) are close-up of waveforms in (**a**,**c**,**e**) showing single EMG response with stimulus artifact indicated by an arrow. (**g**) Locations of EMG electrodes for the measurements in (**a**,**c**,**e**) in the hind limb. Inset shows a picture of a remote diode in free-floating placement on top of the nerve between two platinum based hook electrodes. Scale bar is 1 mm.

**Figure 7:**
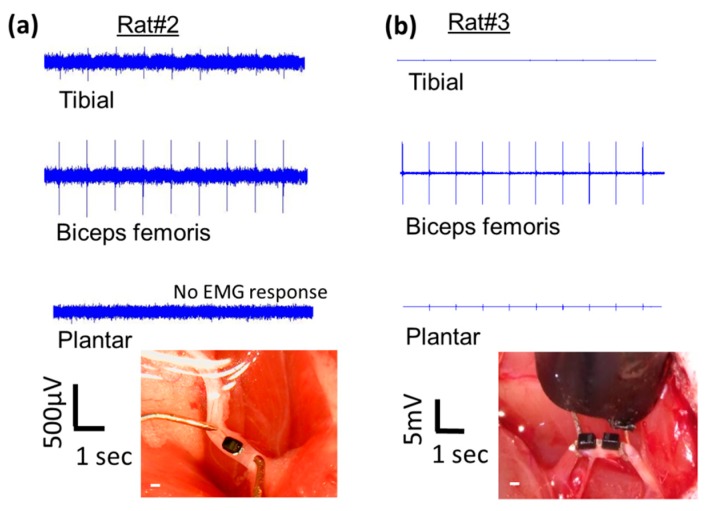
Variable EMG responses from different muscle groups due to (**a**) single, free-floating, SC-79 packaged Skyworks diode stimulated with a high frequency (300 kHz) AC field by platinum hook electrodes and (**b**) two, free-floating SC-79 packaged Skyworks diode stimulated at 1 MHz. The burst repetition rate was 1 Hz for both diode configurations. Relative locations of diodes are shown below the respective EMG responses. Scale bar is 1 mm.

Large (1.5 mm diode–dipole length) diodes placed on nerve tissue rectify the fraction of high frequency currents that pass through them and can cause local neural activation, an example of which is shown in Figure 6. EMG responses recorded via needle electrodes placed downstream in three muscle locations (ankle, biceps femoris, tibialis) (Figure 6g) showed different amplitudes at a stimulus required for 50% of maximum response (Figure 6a–f). The diode was placed close to platinum hook electrodes, as seen in Figure 6. In additional animal experiments (Figure 7a), placement of the diode in a different location between the hook electrodes resulted in variable responses at the three muscle locations (ankle, biceps femoris, tibialis), with the ankle/plantar location showing no EMG response. Increasing the diode length by placing two diodes in tandem resulted in increased EMG amplitudes and a differential activation pattern (Figure 7b). Although the largest responses were seen in the biceps femoris muscle group, the tibial and plantar showed different activation patterns (Figure 6 and Figure 7) depending on location and form factor of diodes.

**Figure 8:**
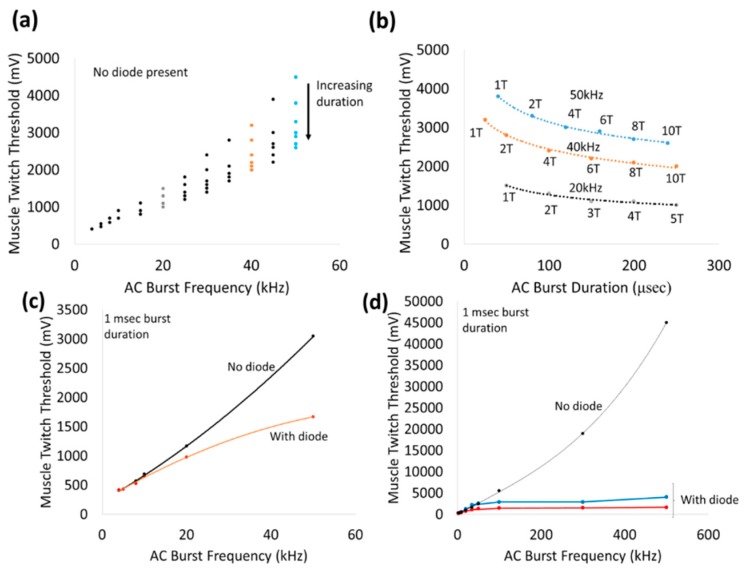
Representative stimulus voltage thresholds for different frequencies of AC stimulation. (**a**) and (**b**) show that the stimulus voltage threshold is dependent on the AC frequency and the burst duration, which is generated by variation in the number of periods (T) or full cycles as seen for 20, 40, and 50 kHz in (**b**). At 20 kHz, increasing burst duration from 50 μsec (1 T) to 1 msec (**c**) decreased the threshold stimulus by 45–55%. The trends observed in the curves in (**b**) are best fit to a power relationship (R^2^ > 0.99). (**c**) The presence of the diode decreases stimulus voltage threshold in a frequency-dependent manner (≥20 kHz). At higher frequencies (**d**), the stimulus voltage threshold increases non-linearly with the input AC frequency in the absence of a diode. The blue (forward bias away from cathode) and red (forward bias toward cathode) lines indicate the presence of a diode in two different orientations.

To better understand the ability to activate remote diodes, a nerve cuff-based platform was utilized. The outer rings of the nerve cuff platform was used to deliver an AC stimulation burst with varying frequencies (0–500 kHz) and durations (33 µsec–1 msec) to an in vivo sciatic nerve preparation with a repetition rate of 1 burst/second in 2 animals. As frequency of the AC stimulation increases, the stimulation drive voltage for muscle twitch threshold increases exponentially as shown in Figure 8a. For increasing burst durations of AC, the threshold for muscle twitch decreases monotonically as shown in Figure 8b. When diodes are wired between two rings closest to the stimulation electrodes (rings ‘2’ and ‘8’) as illustrated in Figure 3b, the stimulus voltage threshold decreases over all frequencies 20–50 kHz as shown in Figure 8c. Interestingly, the stimulus threshold plateaus for frequencies >100 kHz ranging 1.5–5 V stimulus drive voltage as shown in Figure 8d. In contrast, the stimulus threshold in the absence of the diode continues to increase exponentially reaching ~45 V for 500 kHz bursts. This significant decrease in stimulus voltage threshold in the presence of a diode in the current paths in the nerve will be the key property that will help us achieve a spatially selective neural stimulation that is localized in the vicinity of the diodes themselves. Switching the anode–cathode orientation of the diode between the same 2 cuff rings results in only a marginal change in the stimulus voltage threshold as shown in Figure 8d.

The best fit suggests a quadratic relationship (y = 0.1199x^2^ + 28.829x + 371.27, R^2^ > 0.99). However, with the introduction of a diode in the current paths (between rings ‘2’ and ‘8’), the stimulus voltage thresholds are significantly reduced, particularly for frequencies >100 kHz (1.5–5 V drive stimulus). While noting the stimulus is AC, switching the diode orientation toward the cathode (defined as the lead that connects to instrument ground) only changed the stimulus voltage threshold marginally (red versus blue line). Note that the stimulus voltage threshold 500 kHz in the absence of a diode was determined using an additional amplifier to the input signal to achieve high voltages.

### 3.2. Diode–Dipole Length, Relative Position to the Stimulation Electrodes, and Implantation Depth within the Nerve Are Major Factors in Determining Stimulation Thresholds

The stimulus voltage threshold for a visible muscle twitch was assessed as a function of diode–dipole length (based on the setup illustrated in Figure 3b) in 4 additional animals (*n* = 6 sciatic nerves for 20 and 500 kHz and *n* = 5 sciatic nerves for 50 kHz). The average stimulus voltage threshold was inversely proportional to the diode–dipole length. In negative control experiments without diodes, stimulus voltage thresholds were 876 ± 94 mV at 20 kHz and 2.85 ± 0.5 V at 50 kHz. At 500 kHz, the stimulus voltage threshold exceeded instrumentation range. Previous work in Figure 8d suggested the stimulus voltage threshold for 500 kHz to be approximately ~45 V.

**Figure 9:**
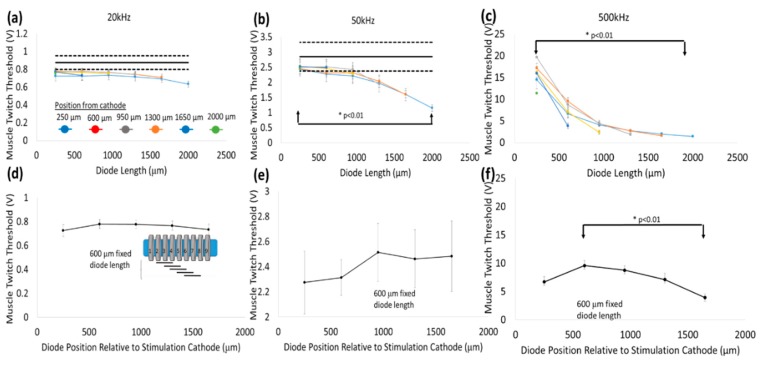
Stimulus voltage thresholds as a function of diode–dipole lengths for different frequencies −20 kHz in (**a**), 50 kHz in (**b**) and 500 kHz in (**c**) and relative position of the diode with respect to the stimulus cathode. Stimulus AC input was delivered on rings ‘1’ and ‘9’ (2.7 mm distance). Stimulus thresholds for conditions with no diodes are shown as the mean (solid black line) with 95% confidence levels (dashed, black lines) in (**a**,**b**). Stimulus voltage thresholds in control experiments with no diodes at 20 kHz (*n* = 6 sciatic nerves, 3 left and 3 right nerves from hind limb) and 50 kHz (*n* = 5 sciatic nerves 2 left and 3 right side) are 876 ± 94 mV and 2.85 ± 0.5 V respectively. At 500 kHz no visible muscle response was observed up to 20 V (instrument limit) (**d**–**f**) show the change in stimulus voltage thresholds due to relative position of the diode with respect to the stimulating electrodes when the diode–dipole length was fixed at 600 μm. The illustrative inset in (**d**) shows the change in position of the fixed diode length relative to the stimulating electrodes. All experiments were conducted with 1 msec AC burst duration. All data are expressed as mean ± SE. Significance was assessed by one-way analysis of variance (ANOVA), followed by Student’s *t*-test (α = 0.01) between the maximum and minimum in (**a**–**f**).

Stimulus voltage thresholds decreased with increasing diode–dipole lengths as shown in Figure 9a–c. At 20 kHz, the change in stimulus voltage threshold with respect to dipole length was less pronounced (~20% decrease at 2 mm diode–dipole length) and was found not statistically significant (one way-ANOVA) as shown in Figure 9a. At 50 kHz, the stimulus voltage threshold for a diode–dipole length of 2 mm was ~55% of that during control AC stimulation (without a diode) and were found to be statistically significant (*p* < 0.01) as shown in Figure 9b. At 500 kHz, the effects of diode–dipole lengths were more pronounced were found to be statistically significant (*p* < 0.01). Even the smallest diode–dipole length (250 μm) at 500 kHz resulted in stimulus voltage threshold that is ~20–50% of that of control experiments with no diode. The larger dipole lengths (>500 μm) resulted in stimulus voltage thresholds that were <10% of that of control experiments with no diodes as shown in Figure 9c.

In addition to the diode lengths, diode position and relative placement with respect to the stimulating electrodes played a role in determining threshold stimulus value. In Figure 9a–c, multiple data points at each diode length correspond to different positions of the diode relative to the stimulus electrode. Figure 9d–f shows a representative example of how stimulus threshold changes as a function of diode position for different frequencies when the diode–dipole length is fixed at 600 μm. A schematic of the relative diode position with respect to the cuff leads is shown in the inset in Figure 9d. At all three frequencies (20, 50, 500 kHz), the proximity of the diode to the stimulus electrodes decreases stimulus threshold, while diode placement toward the center increases the stimulus threshold maximally by ~10% and was not found to be significantly different for 20–50 kHz. At higher diode lengths (>1 mm), the relative change in threshold due to position was minimal. At 500 kHz, the minimum (near stimulation electrodes) and maximum values (closer to center) were found to be statistically significant for diode position in an AC field. It should be noted while the differential trends due to diode position are evident at 500 kHz, there is large variation between samples, suggesting the field lines within the nerve are variable. At 20 and 50 kHz, the contributions of the AC field from the external electrodes towards stimulating the nerve diminish the effect of diode position within the AC field.

**Figure 10:**
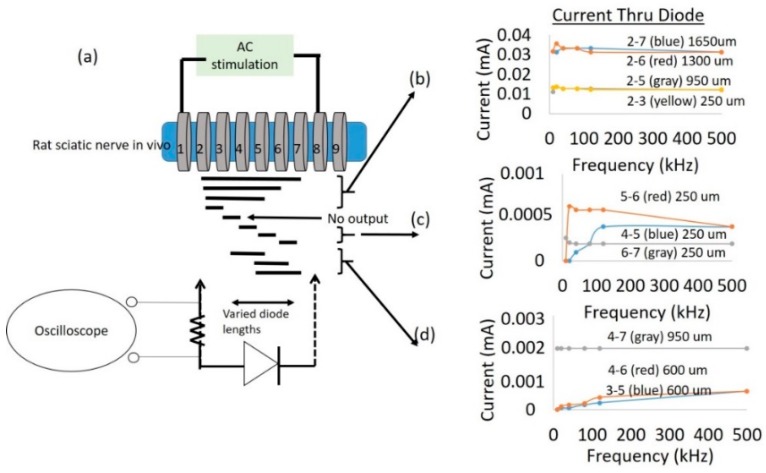
Typical current measurements through the diode for different diode lengths and positions for a 1 V (peak-to-peak amplitude) AC drive voltage. The experimental setup and the different diode lengths and positions that were tested are shown in (**a**). The diode was externally attached to different cuff leads as indicated to emulate different diode length and position relative to stimulating electrodes. (**b**) Larger diode–dipole lengths and placement within 250 µm of a stimulating AC electrode resulted in larger measured currents. (**c**) Shorter diode–dipole lengths of 250 µm and placement >250 µm away from the stimulating electrodes showed 20–60 fold less current through the diode. (**d**) Current measurements for diode–dipole lengths of 600 µm and 950 µm and placement >250 µm away from the stimulating electrodes.

**Figure 11:**
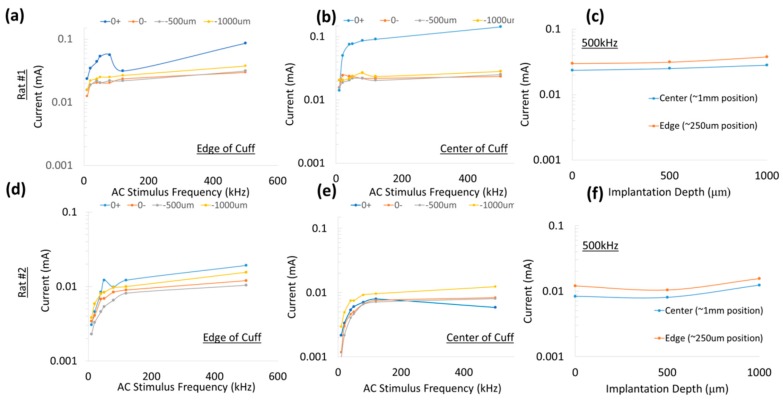
Characterization of current through a remote diode at 3 different implantation depths and 2 different lateral positions along the cuff. All currents were measured in response to a peak-to-peak excitation voltage of 1 V applied across rings ‘1’ and ‘9’ of the cuff electrode. (**a**,**d**) Current through diodes placed at the ‘edge’ of the cuff in 2 different animals and (**b**,**e**) ‘center’ of the cuff for 3 different depths—above the epineurium (0+), just below the epineurium (0−), at 500 μm and 1 mm implantation depths. (**c**,**f**) Representative currents through the diodes at 500 kHz are shown for different implantation depths and position. The highest currents were attained closer to the epineurium (0 or 1 mm depth) of the nerve and closest to the stimulating electrodes.

In an effort to assess the typical currents that flow through the diode, a total of 3 animals were used to test different diode–dipole length configurations in Figure 10 and Figure 11. To better understand the currents that flow through a diode using remote AC stimulating electrodes, current through the diode was measured for different diode lengths and positions (Figure 10a) for a 1 V (peak-to-peak amplitude) AC drive voltage through rings ‘1’ and ‘9’. Interestingly, the current flowing through the diode was generally invariant in the employed frequency range (up to 500 kHz), suggesting that interactions of diode–electrode impedance and tissue impedances were responsible for this effect.

While the experiments in Figure 10 represented currents through ring electrodes with large surface areas, the experimental setup described in Figure 4 measured currents through a ‘remote’ microdiode with a small electrode surface area (cross-sectional face of ~110 µm diameter wire) at various positions and implantation depths. The platinum leads spaced 1 mm apart (for a diode–dipole length of 1 mm) were placed at the “edge” and “center” of the nerve encompassed by the nerve cuff are shown in Figure 11 for 2 animals for a peak-to-peak excitation voltage of 1 V applied across rings ‘1’ and ‘9’ of the cuff electrode. In one of the animals, measured currents through the diode placed on top of the epineurium (0+) was marginally higher than the currents measured from implanted diodes. Similarly, only marginal differences were observed between currents through diodes placed at the “edge” (or closer to the excitation electrodes) versus currents through diodes placed at the “center” of the cuff.

Experiments were also conducted in one animal to test the minimum monophasic current pulse through the diode required to achieve the stimulus current threshold. The stimulus current threshold for the diode was 17 μA at 10 kHz frequency for 1 msec burst duration. Similar experiments at 900 mV drive voltage at 10 kHz frequency with 1 msec burst duration showed that 19.6 μA would be required to reach stimulus threshold. In an additional separate control animal using hook electrodes spaced 3–4 mm apart, 15 µA was required to achieve stimulus threshold at 500 kHz, 500 µsec burst duration. The currents through the remote diode in Figure 10 for the different implantation depths have similar µA range, suggesting remote neurostimulation is feasible for microscale diode implants. Low µA range currents are also reported for stimulators placed close to the nerve [24,25].

### 3.3. Validation of Remote Neurostimulation Using Implanted Mini- and Micro-Diodes Using the Optimal Stimulation Frequency, Diode Dimensions and Placement Determined

**Figure 12:**
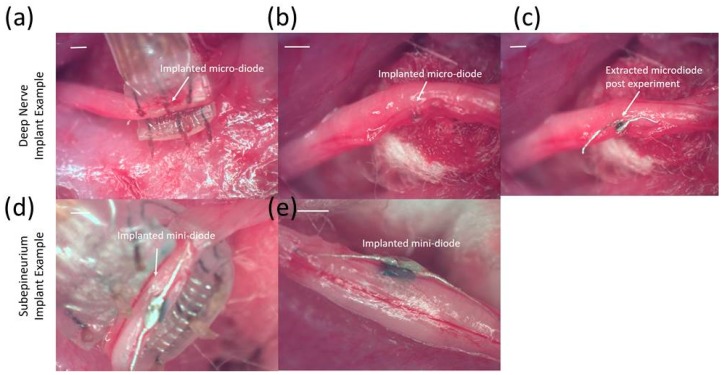
Representative images of implanted micro- and mini-diodes. (**a**–**c**) represent an example of a diode with a 220 μm feature size and lead lengths to match the length of the cuff implanted deep inside the nerve. (**d**,**e**) show an example of a diode with 0.5 mm feature size with 3 mm long leads, the entirety of which is implanted subepineurally.

Remote, free-floating, implanted mini- and micro-diodes with modified lead lengths to match the length of the stimulating nerve cuff (~3 mm) were demonstrated to stimulate the sciatic nerve. Figure 12 shows representative micro-diodes implanted deep into the nerve and hence not visible in the images (Figure 12a,b) and mini-diodes placed subepineurially (Figure 12d,e), where the outer sheath held the diode in place even when subjected to mild shear forces.

**Figure 13:**
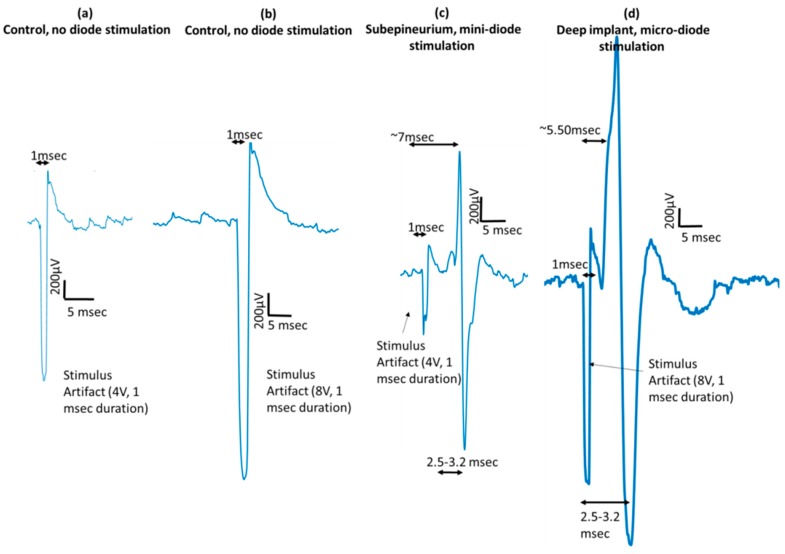
Activation of remote diodes using AC stimulation waveforms induces distinct dose-dependent EMG responses. (**a**,**b**) EMG responses are not observed in the absence of any diodes to AC stimulation of 4 V and 8 V. (**c**) Distinct EMG peak amplitudes are seen ~7 msec after the stimulus artifact in response to activation of diodes placed subepineurially and ~5.5 msec after stimulus artifact in response to activation of diodes placed deep in the nerve (**d**). These representative EMGs were recorded in response to an AC stimulus of 500 kHz frequency with a 1 msec stimulus duration.

When a diode was placed on the nerve or implanted in the nerve between the two stimulating electrodes and activated using the ring electrodes ‘1’ and ‘9’ of the cuff electrode at 500 kHz and 1 msec pulse duration, a visible muscle twitch was visually observed and a corresponding, typically biphasic EMG response was recorded (Figure 13). Representative EMG signals in response to activation of a deeply implanted diode and another subepineurial diode are shown in Figure 13. Control AC stimulations in the absence of any implanted diodes showed only the stimulus artifact, which has a duration of 1 msec. Needles for EMG recordings were placed in digit 5 of the hind paw. A similar range of latencies was seen across 3 implanted animals (5.8 ± 1.3 msec) in response to activation of both subepineurially implanted diodes and diodes implanted deep in the nerve. The EMG waveform has a peak-to-peak duration of 2.6 ± 0.55 msec in all animals.

**Figure 14:**
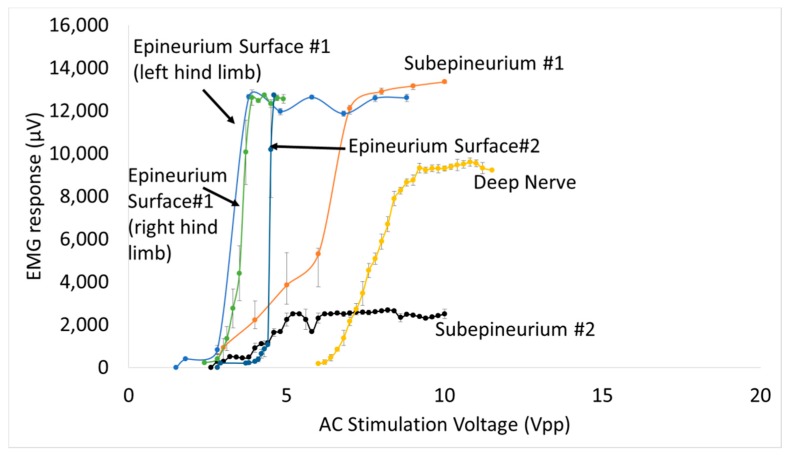
EMG recruitment curves for diodes implanted that are implanted subepineurially (2 animals) and deep in the sciatic nerve (one animal shown). A second animal with a deep nerve implant showed a response only at 20 Vpp (not shown). In addition, EMG recruitment curves of three diodes placed on the sciatic nerve is also shown labeled “epineurium surface #1’ and ‘epineurium surface #2’. Each data point is represented by the mean ± standard deviation of EMG responses to 10 stimulations at a given amplitude.

Muscle EMG recruitment curves for subepineurial diode implants and deep nerve diode implants (*n* = 4 additional animals for all implants) are shown in Figure 14. Subepineurial implants in 2 animals had thresholds in the range ~2.8–3.0 V and had recruitment curves comparable to those obtained for diodes just placed on the nerve implants with 1.5–2.8 V threshold stimulus. It was noted that during the recording of the second recruitment curve for the subepineurial implant #2, the stimulus was selective to movement of only digit 5 suggesting recruitment of localized axonal fibers; whereas with the placement of the diode on the nerve in the same animal or in the case of the subepineurial implant #1 a larger recruitment was seen, causing the whole hind leg to move at higher stimulus voltages. Deep nerve implants in 2 animals had a higher stimulus voltage threshold (6.0 V in one case and 20 V in the second case). The EMG recruitment curve for the first diode implanted deep in the nerve is shown in Figure 14. The recruitment curves for the second implanted diode could not be obtained due to high activation voltages that were needed. EMGs for this animal had a large peak-to-peak amplitude (~12 mV) for the diodes implanted deep in the nerve (data not shown).

## 4. Discussion

The primary goal of this study was to determine the set of stimulation parameters, diode dimensions and placement that would enable microscale, implantable diodes to function as wireless neuromodulators. The working principle was to use the volume conduction properties of tissue as a method of transferring power from non-contacting but nearby electrodes to free-floating diodes placed on or inside the nerves. The initial concept of using a rectifier (germanium diodes with silver leads (1–3 cm long) to stimulate external organs was demonstrated by Palti in 1966 [17]. Recent work reiterated the concept by placing leads from a full-wave bridge rectifying circuit prototype (eAxon) into selected muscle fibers that are stimulated using a 1 MHz sinusoidal input [18]. In this study, we demonstrated remote neurostimulation using microscale, silicon diodes directly implanted in the nerve in at least 13 animals (Figure 6, Figure 7, Figure 8, Figure 9, Figure 12 and Figure 13). This approach allowed neurostimulation without wires traversing the epineurium to contact electrodes. Thus, there is a wireless bridging of the last millimeter of distance between the local environment outside of the nerve body and its interior. Using different feature sizes (1.5 mm, 0.5 mm, and 0.22 mm) off-the-shelf, commercially-available, Schottky diodes (Skyworks 7630), we assessed the parameters of the external stimulating AC signal (such as frequency 10–500 kHz, drive voltages, and currents), diode dimensions and relative position of the diode with respect to the external AC stimulating electrodes that would be required to achieve wireless neurostimulation for microdiodes implanted in the sciatic nerve. We found AC stimulating frequency and diode length to be major factors in diode performance in vivo, followed by proximity of the diode to the stimulating electrodes and implantation depth.

Application of 1 msec bursts of zero-offset, sinusoidal AC waveforms via a nerve cuff platform by itself stimulated the sciatic nerve in a frequency-dependent manner from 10–500 kHz (Figure 8). The non-linearly increasing thresholds required to achieve a visible muscle twitch at higher frequencies can perhaps be explained by earlier observations of classic strength–duration relationships for nerve stimulation. Such strength–duration curves for nerves have demonstrated a non-linear, hyperbolic relationship between strength and duration required to achieve threshold. High frequencies correspond to lower durations and hence threshold for AC stimulation of nerves can be expected to increase non-linearly with frequency. In addition, high frequency AC stimulation that exceed the kinetics of the ion channels in the cell membrane, will result in significantly higher voltage thresholds (such as ~45 Vpp at 500 kHz). In fact, pure sinusoidal AC continuous waveforms up to 40 kHz have been used effectively in nerve conduction block applications, such as relief from phantom limb pain [26,27,28].

Placement of a wireless, remote diode in AC electric field is expected to fully or partially rectify the input sinusoidal wave and generate a DC component that is proportional to input Vrms and large enough to stimulate a nerve. Above 20 kHz, the addition of a wireless diode between the stimulus electrodes achieved increasing reductions in the stimulus voltage threshold. For instance, the stimulation voltage thresholds at 20 kHz and 50 kHz were ~700 mV and ~1.5 V with a wireless diode compared to ~900 mV, ~3 V without diode, respectively. Beyond 100 kHz, the stimulus voltage threshold plateaued (<5 V) and was fairly independent of frequency. We speculate that between 20–100 kHz, the rectified signals of the diode augmented the neurostimulation of the applied external AC signal in achieving the threshold. Below 20 kHz, the augmentation effect of the diode presence was not significant, suggesting the neurostimulation was dominated primarily by the external AC stimulation. Frequency-dependent effects are not expected from diodes since current-voltage (I-V) characterization of modified diodes show similar diode threshold values and rectification properties across a range of frequencies (10 kHz–1 MHz, Figure 2).

In this study, the measured currents through the diode were at least 100-fold less (Figure 10) than the currents through the rings ‘1’ and ‘9’ of the cuff electrode (Figure 5), suggesting high levels of volume conduction loss. Increasing the diode–dipole length from 250 µm to 2 mm reduced the threshold by ~10–20% and ~55% at 20 kHz and 50 kHz, respectively, as shown in Figure 9. Significant improvements in stimulus voltage thresholds were seen for diode–dipole lengths >1 mm at 50 kHz, while the effect of diode length was only marginal at 20 kHz. The effects of diode–dipole lengths were more pronounced for 500 kHz stimulation frequency. The stimulus voltage threshold lowered 10-fold at 500 kHz as the diode–dipole length changed increased from 250 µm to 2 mm. The smaller diode–dipole lengths have relatively lower energy transfer efficiency due to less volume-conducted currents being intercepted by diode–electrodes. At high frequencies, only the current through the diode that is rectified would be useful for neurostimulation. The results are in agreement with [23], who theorized that diode designs with long, thin geometry that maximize separation distance with short electrode leads would have maximum energy transfer efficiency. Sahin et al. [22] showed that separation distance of remote electrodes more than two times the diode anode–cathode separation distances (or diode–dipole length) entails high volume conductor losses. The stimulating electrode in the cuff were separated by 2.7 mm and, indeed, at 500 kHz where the augmentation of the diode would be most dominant, a diode–dipole length of >1 mm resulted in the lowest voltage thresholds.

For a fixed diode–dipole length, increasing the diode proximity to the stimulating electrodes reduced the stimulus voltage threshold value by ~10% compared to positions more central between the stimulating electrodes (Figure 9d,e). Measured currents through a diode reduced 20–60 times when the diode was placed >250 µm away from the stimulating electrode (Figure 10). A remote diode with a smaller contact surface area also reduced by up to 2-fold toward the central position between two stimulating ring electrodes and toward ~500 µm implantation depth (Figure 11). In the case of implanted microdiodes, a large contact surface area between the anode/cathode and the tissue is attained via an additional 1 mm extension of bare, uninsulated platinum wire (total length of the microdiode and wire extension ~3 mm). The larger contact surface area allowed for a lower interfacial impedance and hence a more conducive current path compared to the typically higher tissue impedance surrounding the implant. The feasibility of obtaining recruitment curves from implanted microdiode stimulators (Figure 14) confirmed that having lower contact impedances is an important design parameter in addition to diode–dipole length and placement in the AC field.

An interesting point was that diodes placed on the nerve and diodes embedded just underneath the epineurium had similar threshold values, suggesting the epineurium did not impede in the energy transfer between the stimulating electrode and the diode. It is well known that at high frequencies (such as 500 kHz) the impedance of electrodes placed above the epineurium and those implanted just beneath in the nerve (sub-epineurium) would converge, essentially eliminating any impedance mismatches for energy transfer. However, implants placed deeper in the peripheral nerve tissue would be expected to have a higher threshold due to higher tissue path impedance. Indeed when comparing EMG recruitment curves of diodes implanted deep in the nerve and that of subepineurial diodes for similar diode lengths at 500 kHz input frequency (Figure 14), the stimulus voltage threshold increased by 2–3 fold from 2.8–3 V to 6 V. In fact, one diode implanted deep in the nerve required a stimulus voltage threshold of 20 V, suggesting steep recruitment curves (data not shown). It should be noted that the minimum current needed to achieve the threshold were similar for a monophasic, square pulse with a 1 msec duration (17 µA) compared to the minimum current through a diode at the threshold (19.6 µA). Therefore, deep implants require more drive to achieve similar performance. It should be emphasized the repeatability and robustness of the diode placement would be an important experimental variable in potential application of this kind of neurostimulation strategy. The repeatability/robustness of the nerve responses from the diodes from trial to trial is governed partly by surgical technique and animal-to-animal variations in electrophysiological response. The primary focus of this study was not to characterize known biological responses to pulsed monophasic but rather the ability to stimulate them remotely by electric field manipulation. Strategies to optimize positioning and manipulation of diode lengths will be needed in the future for modulation of deep fibers. Volume conduction models that assume homogenous and isotropic tissue properties with uniform conduction would predict the highest threshold to be at the midpoint. However, the data in Figure 8d–f suggested that while stimulus thresholds trended higher in the region between the edges of the stimulus electrodes, there was a large variance in the exact position where least current and higher thresholds occur. Considering that tissue properties have in reality more inhomogeneous composition [29], and the nerve itself has a non-spherical, oblong geometry, better mapping of conduction properties inside the nerve would be needed in the future for the optimal placement of diodes. It should be noted that variance in current properties outside the nerve epineurium (Figure 10) was possibly due to the presence and effective concentrations of body fluids over the time course of the experiment. It should be made clear that the study is not proposing the placing of commercial diodes in nerves, since even at the smallest feature size of 220 µm, they may cause significant tissue damage due to relative tissue motion. However, assessment of the biocompatibility of the current remote diode–dipole chips is beyond the scope of this work since the primary motivation here was to investigate the possibility of inter-neurally, implantable microstimulators to be remotely activated. The potential advantages of this approach are selective targeting and decoupling of the physical wire connectivity to mitigate the relative motion between implanted devices and the nerve tissue. The disadvantages are relatively higher currents applied to nerve cuffs (currents in the cuffs are in the order of a few mAs to 10’s of mA which are ~100 times higher than currents through the remote diodes) and limitations in placement of multiple diodes due to the need for diode electrode lengths in the order of 1 mm.

This often affects the performance of tethered implants in terms of energy usage, targeting precision and optimal performance since it may require frequent recalibration. This work did not examine issues of nerve damage due to diode placement. However, we note that to mitigate the tissue damage due to diode implant itself, design modifications such the use of materials that are mechanically matched to the tissue, flexible designs and miniaturization may be used to improve chronic functional performance.

## 5. Conclusions

We observe that small 220 µm, free-floating, Schottky micro-diodes placed free-floating inside a rat sciatic nerve can reduce thresholds and stimulate action events using high frequency AC bursts with 1 msec duration. The advantage is that no chronic trans-epineurial wires to individual nerve fibers are needed to locally stimulate fibers. This work suggests that free-floating diodes placed internal to the nerve in combination with nerve cuffs thus can act similarly to penetrating electrodes in achieving selective (focal) sites of nerve stimulation but without penetrating wires implanted intraneurally. Experiments show, however, strong sensitivity to diode–dipole length with the minimum values of in the order of 1 mm, frequency of AC carriers, limits on the distance of diodes from stimulation electrodes, and thus implantation depths at practical AC drive currents. This places significant limits on extrapolations of this approach to multiple channels. Currents through implanted diodes within the nerve suffer large attenuations (~100 fold) compared to AC burst currents requiring relatively high (1–2 mA) drive currents. Muscle EMG curves with implanted free-floating diodes are intrinsically steep and get steeper as a diode is placed at increasing depths away from external AC stimulating electrodes.
